# Sleep Education in Otolaryngology Residency Programs: Trends Over the Past Decade

**DOI:** 10.1002/ohn.70008

**Published:** 2025-08-28

**Authors:** Nicole Molin, Elliott M. Sina, Erin Creighton, Praneet C. Kaki, Maurits Boon, Colin Huntley, Cristina M. Baldassari

**Affiliations:** ^1^ Department of Otoloaryngology Head and Neck Surgery and Sleep Medicine Ear Nose and Throat Consultants of Nevada Las Vegas Nevada USA; ^2^ Sidney Kimmel Medical College Thomas Jefferson University Philadelphia Pennsylvania USA; ^3^ Department of Otoloaryngology Head and Neck Surgery and Sleep Medicine Arkansas Otolaryngology Center Little Rock Arkansas USA; ^4^ Departments of Otolaryngology & Sleep Medicine Thomas Jefferson University Hospital Philadelphia Pennsylvania USA; ^5^ Department of Otolaryngology Eastern Virginia Medical School Norfolk Virginia USA; ^6^ Department of Sleep Medicine Children's Hospital of the King's Daughters Norfolk Virginia USA

**Keywords:** obstructive sleep apnea, residency education, sleep medicine, sleep surgery

## Abstract

**Objective:**

We aim to assess current trends in sleep education in otolaryngology residency training programs in the United States.

**Study Design:**

Cross‐sectional survey.

**Setting:**

Otolaryngology residency programs in the United States.

**Methods:**

Survey sent to otolaryngology program directors. Responses compared to results of prior sleep education surveys.

**Results:**

In total, 31 program directors responded (response rate 29%). In total, 93% reported having faculty with clinical time dedicated to the treatment of sleep disorders, an increase from 58% in 2011. However, only 29% reported having faculty with board certification in sleep medicine. In total, 71% have at least 0.6 months of cumulative training time related to sleep medicine, a significant increase from 34% in 2011 (*P* < .01). In total, 61% report at least 5 hours of didactics on sleep medicine in their curriculum compared to 16% in 2011 (*P* = .01); 77% of programs provide education on the interpretation of home polysomnograms (48% 2011) (*P* = .02), while 83% provide residents with training on interpreting in‐laboratory polysomnograms (2011 92%) (*P* = .47).

**Conclusion:**

The current study suggests positive growth with regards to sleep education in otolaryngology residency training over the past decade. Responding programs report an increase in faculty providing sleep medicine treatment, an increase in exposure to home polysomnography interpretation, and an increase in sleep medicine‐related didactic hours. The number of programs with faculty subspecialized in sleep medicine remains low. Although resident exposure to sleep medicine education is improving, additional educational opportunities to enhance resident physicians' sleep education experience would likely be beneficial.

Obstructive sleep apnea (OSA) is a disorder characterized by repetitive upper airway collapse, causing intermittent episodes of nocturnal airflow obstruction. The estimated prevalence of OSA is 9% of women and 24% of men.^1^ Primary treatment for OSA is continuous positive airway pressure (CPAP); however, this treatment is limited by low adherence to therapy.^2^ This creates an increasing need for CPAP alternative therapy, which includes upper airway surgery (UAS).

Otolaryngologists play an essential role in the management of patients with OSA, as they are the primary specialists involved in performing drug‐induced sleep endoscopy and UASs for those intolerant to CPAP. It is important that otolaryngologists are familiar with the evaluation and treatment of OSA patients, as a recent guideline recommends that patients who are unable to tolerate CPAP be considered for referral to discuss alternative treatments.^3^ As the field grows, the number of physicians applying to sleep medicine fellowships from eligible specialties is increasing.^4^ Applicants come from numerous different specialties, including pulmonary, internal medicine, family medicine, pediatrics, otolaryngology, neurology, and psychiatry, among others. The number of accredited sleep medicine fellowship programs offering training in sleep surgery in addition to the standard sleep medicine curriculum is growing.^4^ Of note, sleep surgeries are also performed by general otolaryngologists who have not completed a formal sleep fellowship. With that, sleep medicine and sleep surgery exposure and education within otolaryngology training programs become exceedingly important, and the American Board of Otolaryngology (ABOTO) includes this as part of the mandated curriculum.^5^

Multiple studies have evaluated sleep education within otolaryngology residency programs over the past decade through survey analysis. There appears to be a great deal of variability in resident training between programs when it comes to sleep surgery and sleep medicine exposure.^6^, ^7^, ^8^, ^9^ When comparing trends over time, exposure to oropharyngeal procedures seems to be increasing, with continued lower exposure to hypopharyngeal procedures.^7^ Programs express interest in expanding residents' exposure to sleep medicine and sleep surgery,^6^ and most felt their program would benefit from a dedicated sleep surgeon.^8^ When multiple specialties were surveyed about sleep education within their residency program, most trainees pursuing sleep medicine fellowships were from pulmonary critical care or neurology, and those two specialties also reported the most hours of sleep didactics in their curriculum.^9^

As the field of sleep surgery continues to evolve, the objective of this study was to assess current trends in sleep education within otolaryngology residency training programs across the United States. We sought to determine whether exposure to clinical sleep medicine, sleep surgery, and sleep education within otolaryngology residencies has advanced compared to prior survey studies from 2011^6^ and 2017.^8^

## Methods

### Study Design

Approval was obtained by the institutional review board (IRB) at Eastern Virginia Medical School. The authors developed a survey modified with permission from one used by Shen et al^6^ to allow for comparison of trends over time. The current survey and previous versions have not been validated. Survey questions are detailed in Supplemental Material, available online. The survey included questions related to sleep education within otolaryngology resident programs, focused primarily on adult sleep medicine education. Questions were kept consistent with those from the 2011 study^6^ to allow for comparison, with some new questions added to reflect changes in the field. One of the questions added to the current survey asked about resident exposure to modified uvulopalatopharyngoplasty (UPPP) in addition to traditional UPPP to reflect advances in palate surgery, such as that described by Pang and Woodson.^10^ The current survey data were collected from October to December 2023. The survey was administered to program directors of allopathic and osteopathic otolaryngology programs in the United States with available email addresses, which was 106 program directors. The number of ACGME‐approved otolaryngology training programs in 2022 to 2023 was 131 as reported by the American Academy of Otolaryngology–Head and Neck Surgery (AAO‐HNS).^11^ An email was sent that included a link to the survey, which was accessed via RedCap. A total of two additional email reminders were sent to participants via RedCap to complete the survey following the initial correspondence.

The survey included questions about program demographics, dedicated time of faculty to sleep medicine/surgery, residents' exposure to various sleep surgeries, amount of didactics dedicated to sleep medicine/surgery, and overall satisfaction with the current sleep education within their program. The survey responses remained anonymous.

### Statistical Analysis

Data were analyzed using social science statistics,^12^, ^13^ clinical calc LLC,^14^ and RStudio. A power analysis was performed to determine the required sample size, assuming a medium effect size, alpha of .05, and desired power of 0.8. The required sample size calculated was 84 (42 per group).

Due to the low sample size of the current study and multiple variables with less than five responses, a decision was made to utilize Fisher's exact testing for analysis. *P* values were rounded to the nearest 100th decimal. Data from the current survey were compared to surveys from prior studies, Shen et al, which included surveys from 47 programs, and Gouveia et al, which included surveys from 46 programs, to assess trends.^6^, ^8^ Significance was set to an *α* level = .05 for all tests. Certain variables from the current study were compared only to the 2011 study^6^ due to those variables not being in the published 2017 survey.^8^

## Results

From a total of 106 otolaryngology program directors contacted, 31 responded and agreed to complete the survey (response rate 29%). Residency program characteristics are summarized in Table 1 from the 2011 study,^6^ 2017 study,^8^ and the current study.

**Table 1:** Otolaryngology Residency Program Characteristics From Current 2023 Survey of Program Directors Compared to 2011^6^ and 2017^8^ Surveys

	2011	2017	2023	*P* value 2011 versus 2023	*P* value 2017 versus 2023
No. of residents in each postgraduate year; n (%)					
1‐2	16 (34.8)	13 (28.3)	11 (33.3)	1.00	.61
3‐4	27 (58.7)	25 (54.3)	16 (48.5)	.64	.82
5‐6	3 (6.5)	8 (17.4)	4 (12.1)	.68	.75
No. of clinical faculty members; n (%)					
0‐10	18 (37)	9 (19.6)	3 (9.7)	.01*	.34
11‐20	18 (38.1)	21 (45.7)	17 (54.8)	.16	.64
>20	11 (23.9)	13 (34.8)	11 (35.5)	.31	.80
Is the sleep medicine division a part of the otolaryngology department; n (%)					
Yes	2 (4.4)	11 (23.9)	7 (22.6)	.027*	1.00
No	38 (84.4)	35 (76.0)	18 (58.1)	.016*	.13
Do not have sleep medicine division	5 (11.1)	N/A	6 (19.4)	.34	N/A

* *P* < .05.

### Characteristics of Sleep Faculty

Characteristics of faculty's sleep medicine/surgery practice are summarized in Table 2 from the 2011 study,^6^ 2017 study,^8^ and current study. From the current study, 93.5% of programs reported having faculty with any amount of time dedicated to sleep practice which was higher than reported in 2011 (58.7%, *P* < .01) and 2017 (67.4%, *P* = .01). Only 6.5% of programs reported not having any faculty with time dedicated to sleep; significantly less than reported in 2011 (41.3%, *P* < .01) and 2017 (32.6%, *P* = .01). In total, 29% of programs reported having faculty board certified in sleep medicine, which was not statistically significantly different than rates in 2011 (42.3%, *P* = .27) and 2017 (32.6%, *P* = .81).

**Table 2:** Otolaryngology Residency Program Characteristics of Sleep Faculty From This Current 2023 Survey of Program Directors Compared to 2011^6^ and 2017^8^ Surveys^a^

	2011	2017	2023	*P* value 2011 versus 2023	*P* value 2017 versus 2023
Program has faculty with any time dedicated to sleep; N (%)					
Yes	27 (58.7)	31 (67.4)	29 (93.5)	<.01*	.01*
No	19 (41.3)	15 (32.6)	2 (6.5)		
No. of faculty members who have clinical time dedicated to adult sleep medicine/surgery; n (%)					
0	19 (41.3)	15 (32.6)	2 (6.5)	<.001*	.01*
1	17 (37.0)	19 (41.3)	10 (32.2)	.81	.48
2	6 (13.0)	8 (17.4)	11 (35.4)	.026*	.11
3	3 (6.5)	3 (6.5)	6 (19.3)	.15	.15
4	0 (0)	0 (0)	1 (3.2)	.40	.40
5	1 (2.2)	1 (2.2)	1 (3.2)	1.00	1.00
Program has faculty who spend >50% of clinical time dedicated to sleep					
Yes	11 (23.4)	16 (34.8)	5 (17.8)	.77	.18
No	35 (76.6)	30 (65.2)	23 (82.1)		
Program has faculty who are board‐certified in sleep medicine					
Yes	11 (42.3)	15 (32.6)	9 (29)	.27	.81
No	14 (53.8)	31 (67.4)	22 (71)		
No. of faculty members who are board‐certified in sleep medicine by the American Board of Medical Specialties					
0	14 (30)	N/A	22 (71)	.27	N/A
1	10 (21)		4 (12.9)	.03*	
2	1 (4)		5 (16.1)	.21	
Setting of resident's exposure to adult sleep medicine/surgery					
University hospital	41 (95.3)	N/A	24 (77.4)	.36	N/A
Veterans hospital	20 (46.5)		7 (22.6)	.08*	
Private hospital	12 (27.0)		8 (25.8)	1.00	
Outpatient surgery center	N/A		10 (32.3)	N/A	
Our residents do not have sleep medicine/surgery training	N/A		2 (6.5)	N/A	

^a^ N/A indicates that specific variable was not collected in the 2011^6^ and/or 2017^8^ study.

* *P* < .05.

### Characteristics of Residents' Sleep Medicine/Surgery Training

In total, 6.5% of programs reported residents not having exposure to sleep medicine/surgery training (Table 2). Residency training characteristics are summarized in Table 3 from the 2011 study^6^ and the current study. The 2017 study did not report on these characteristics. Compared to 2011, more programs reported >5 hours per year of sleep didactics (61% vs 16%) (*P* = .01), cumulative training time of at least 0.6 months (71% vs 34%) (*P* < .01) and exposure to home sleep study (HST) report interpretation (61% vs 37%) (*P* = .04). Based on the current study, 32% of programs reported having a resident accepted into a sleep fellowship in the past 10 years. This query was not assessed in prior studies.

**Table 3:** Otolaryngology Residency Program Characteristics of Residents' Sleep Training From Current 2023 Survey of Program Directors Compared to 2011^6^ Survey^a^

	2011	2023	*P* value
Hours of didactics dedication to sleep			
>5 h/y	8 (16.0)	19 (61)	.01*
<5 h/y	39 (84.0)	11 (35)	
Interpretation of home sleep studies			
Report only	17 (37.2)	19 (61.3)	.04*
Original data and report	6 (11.6)	5 (16.1)	.75
None	24 (51.2)	7 (22.6)	.02*
Interpretation of in‐lab sleep study			
Report only	30 (64.3)	17 (54.8)	.48
Original data and report	13 (28.6)	9 (29.0)	1.00
None	4 (7.1)	5 (16.1)	.47
Residency training in analyzing CPAP efficacy			
Yes	10 (21.0)	8 (25.8)	.78
No	30 (63.0)	23 (74.2)	.46
Unknown	7 (16.0)	0	.038*
Cumulative training time > 0.6 mo			
Yes	16 (34)	22 (71)	<.01*
No	31 (65)	4 (13)	<.01*
N/A	N/A	5 (16)	N/A
Program has had resident accepted into sleep fellowship in the past 10 y			
Yes	N/A	10 (32.2)	N/A
No		21 (67.7)	

Abbreviation: CPAP, continuous positive airway pressure.

^a^ These variables were not available from the 2017^8^ study and therefore not included in this analysis.

* *P* < .05.

### Residents' Exposure to Various Sleep Surgeries

Reported residents' exposure to various sleep surgeries from the 2011 study^6^ and current study is reported in Table 4. This was not reported in the 2017 study.^8^ Based on the current study, all program directors reported resident exposure to tonsillectomy/adenoidectomy, 93.5% reported exposure to hypoglossal nerve stimulation (HGNS), 54% reported exposure to traditional UPPP, 80% reported exposure to modified UPPP, 29% to tongue base suspension, 25% to hyoid suspension, 19.4% to midline glossectomy, 19.4% to genioglossal advancement, and 25.8% to maxillomandibular advancement (MMA). The 2011 study^6^ did not report on HGNS or modified UPPP. Comparatively, the current study had fewer program directors report residents' exposure to traditional UPPP (54% vs 100%, *P* < .01), hyoid suspension (25% vs 51.2%, *P* = .04), and midline partial glossectomy (19.4% vs 60.5%, *P* < .01). The current study had more program directors report residents' exposure to MMA (25.8% vs 7%, *P* = .02).

**Table 4:** Otolaryngology Residency Program Characteristics of Residents' Exposure to Various Sleep Surgeries From Current 2023 Survey of Program Directors Compared to 2011^6^ Survey^a^

Surgery	2011	2023	*P* value
T&A	100%	100%	1.0
Hypoglossal nerve stimulation (HGNS)	N/A	93.5%	N/A
Traditional UPPP	100%	54%	<.01*
Modified UPPP	N/A	80%	N/A
Tongue base suspension	39.5%	29%	.34
Hyoid suspension	51.2%	25%	.04*
Midline partial glossectomy	60.5%	19.4%	<.01*
Genioglossal advancement	25.6%	19.4%	.59
Maxillomandibular advancement (MMA)	7%	25.8%	.02*

Abbreviations: T&A, tonsillectomy and adenoidectomy; UPPP, uvulopalatopharyngoplasty.

^a^ These variables were not available from the 2017^8^ study and therefore not included in this analysis.

* *P* < .05.

### Satisfaction of Sleep Training

**Figure 1:**
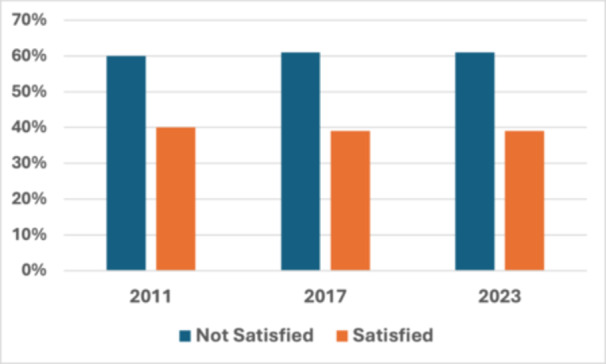
Program directors' satisfaction with sleep education in their residency program: percentage of otolaryngology program directors that reported being satisfied with sleep education in their residency program in the current study compared to findings from survey studies from 2011^6^ and 2017.^8^

Satisfaction with sleep training in residency programs based on responses from the 2011 study,^6^ 2017 study,^8^ and from the current study is displayed in Figure 1. Answering fairly or not satisfied was considered “not satisfied,” answering very or extremely satisfied was considered “satisfied.” The number of program directors that reported being satisfied in the current study was 39%; this was similar to data reported in 2011 (40%, *P* = 1.00) and 2017 (39%, *P* = 1.00).

## Discussion

The current study surveyed 31 otolaryngology program directors on the topic of sleep education within their residency program and was subsequently compared to prior survey studies on the topic. Our data identified several positive trends over the past decade and highlighted multiple areas for improvement.

There appears to be a positive trend with regards to programs having faculty with dedicated time to sleep medicine/surgery. Since 2011, there were significantly more program directors reporting faculty with any time dedicated to practicing sleep, 58% compared to 93% in the current study.^6^ The majority of program directors reported having at least two faculty, one reported four, and one even reported five faculty with time dedicated to sleep medicine/surgery. Only 18% of programs, however, reported having faculty that dedicate more than 50% of clinical time to sleep, and only 29% reported having at least one faculty that is board‐certified in sleep medicine. Although this indicates a positive trend overall, this leaves many programs without exposure to sleep boarded faculty and two programs who reported having no faculty with time dedicated to sleep practice and therefore less clinical exposure for residents. Of note, a recent study identified and surveyed 26 surgeons who completed a sleep‐focused fellowship from 2017 to 2023; 89.5% reported being in academics. This suggests that the number of programs with sleep‐boarded faculty reported in the current study may be an underestimation and warrants further investigation.^13^ As the ABOTO now includes sleep medicine/surgery as part of the mandated curriculum,^5^ this signifies the need for additional resources that can be offered to such programs to augment residents' sleep education during residency training.

Even for those programs that incorporate sleep education, there remains a significant level of variability. Since 2011, there seems to be a notable uptrend in hours of sleep didactic.^6^ However, 11 programs still reported having less than 5 hours per year. The average hours of sleep didactics for all specialties that are involved in sleep medicine is 4.75 hours per year; however, specialties that report the highest number of sleep fellowship applicants (pulmonary critical care and neurology) report the highest number of sleep didactic hours of any specialty: 7.4 and 5.8 hours per year, respectively.^9^ Increasing didactic hours may therefore be beneficial. It is important to note that with a finite amount of time for yearly resident didactics, adding sleep medicine didactics would take away from other topics and specialties of equal importance. Therefore, there may be an opportunity to both standardize and increase exposure to sleep medicine education through the recently developed online Otolaryngology Residency Core Curriculum from the American Academy of Otolaryngology.

The current study suggests a positive trend with exposure to HST interpretation, both the report and the original data. The reported exposure to in‐lab sleep study report interpretation slightly declined since 2011; however, this was not statistically significant, and exposure to original data from in‐lab sleep studies remained roughly similar.^6^ This trend towards HST exposure parallels clinical trends towards more HSTs being used for the diagnosis of OSA as opposed to in‐lab sleep studies. In total, 23% of programs reported no exposure at all to HST interpretation, and 16% reported no exposure to in‐lab sleep study interpretation, further highlighting the need for external resources for residents in these areas.

Residents' exposure to various sleep surgeries remains highly variable. The majority of programs reported exposure to HGNS and modified UPPP. There was a decrease in exposure to other tongue base procedures and traditional UPPP, which correlates with practice patterns within the field of sleep surgery, as modified palatal surgeries and HGNS are performed with increasing frequency. There was an increase in exposure reported to MMA, though overall, the programs reporting exposure still remained low at 26%.

The overall satisfaction of program directors with the current state of sleep education within their residency remains low, with only 39% of program directors reporting satisfaction. With the growth of the sleep medicine/surgery field and the variability that was evidenced in the current survey study, this low satisfaction is not entirely surprising. Even with the recent growth, the field of sleep surgery/medicine overall remains small. Programs with less resources for sleep education could greatly benefit from additional resources, including a standardized sleep medicine/surgery curriculum for otolaryngology residents.

There were multiple limitations of this study. The most significant limitation being the low response rate, as is the case with many survey studies, which limits the generalization of results and introduces potential bias. In addition, as surveys from the current study were anonymous, it is unclear whether the same program directors responded to both surveys. Of note, there were a subset of otolaryngology program directors without up‐to‐date or available emails, and therefore these programs were not sent the survey. There may also be response biases with programs with less sleep surgery exposure failing to respond. Despite these limitations, we believe this study still provides valuable information about variability among programs with regards to sleep education. This study was also limited because the response was from a single individual within the program. Although this did limit programs from being represented more than once, surveying multiple people within a program could provide different and equally important perspectives. There may also be a limitation to the survey question asking, “Is the sleep medicine division a part of the otolaryngology department at your institution?” The number of program directors answering yes to this question seems higher than what has been observed, which may be related to respondents' interpretation of the question.

Future studies, including a larger‐scale survey of more diverse participants, including surveying residents, may prove beneficial in further understanding areas of growth and needs for improvement. To circumvent the limitation of low response rates with survey studies, a query of program websites is an alternative way to evaluate this topic. Information from websites, such as the number of faculty listed with dedication to sleep medicine/sleep surgery and the number of faculty with board certification in sleep medicine, could be collected to provide further insight into this topic. Another opportunity for future study design is to obtain otolaryngology residency program directors' input on the survey questions, findings, and conclusions of the current study. Implementing variables such as a national curriculum or other resources for programs to augment sleep education within otolaryngology residencies and monitoring the impact on education and program directors' satisfaction will also be important.

## Conclusion

The current study suggests positive growth with regards to sleep education in otolaryngology residency training over the past decade. Responding otolaryngology residency program directors report an increase in faculty providing sleep medicine treatment, an increase in exposure to HST interpretation, as well as an increase in sleep medicine‐related didactic hours. The number of otolaryngology residency programs with faculty subspecialized in sleep medicine remains low, and a small number of program directors reported no resident exposure to sleep medicine. Although otolaryngology resident exposure to sleep medicine education is improving, there remains a need for additional educational opportunities to enhance resident physicians' sleep education experience.

## Author Contributions

**Nicole Molin**, study design, data collection, data interpretation; manuscript writing; **Elliott M. Sina**, data collection, data interpretation, manuscript writing; **Erin Creighton**, data collection, data interpretation, manuscript writing; **Praneet C. Kaki**, data interpretation, manuscript writing; **Maurits Boon**, study design, data collection, data interpretation, manuscript writing; **Colin Huntley**, study design, data collection, data interpretation, manuscript writing; **Cristina M. Baldassari**, study design, data collection, data interpretation, manuscript writing.

## Disclosures

### Competing interests

Nicole Molin: none. Elliott M. Sina: none. Erin Creighton: none. Praneet C. Kaki: none. Maurits Boon: chief medical officer Nyxoah. Colin Huntley: consultant for Nyxoah and Inspire. Cristina M. Baldassari: consultant for Nyxoah; grant funding from Nyxoah and Inspire.

### Funding source

None.

## Supporting information

Supporting Information.
