# Supplementary material for: Sleep Education in Otolaryngology Residency Programs: Trends Over the Past Decade
Source: Otolaryngol Head Neck Surg. 2025 Aug 28;173(6):1500–6. doi: 10.1002/ohn.70008 (PMC12661478; doi:10.1002/ohn.70008)
Supplement: Supplementary file 1 — Supporting Information. [file OHN-173-1500-s001.docx]

**Supplemental Materials:** Survey Questions

I consent to participating in this survey

1, Yes

2, No (if you do not consent you may exit the survey at this time)

How many of residents per year does your residency program accept?

1-2

3-4

5-6

N/A

How many clinical faculty members are a part of your residency program?

0-5

6-10

11-15

16-20

>21

N/A

Is the sleep medicine division a part of the Otolaryngology department at your institution?

Yes

No

We do not have a sleep medicine division

N/A

In which of the following settings do residents receive adult sleep medicine/surgery training?

(more than one answer allowed)

University hospital

Veterans hospital

Private hospital

We do not have sleep medicine/surgery training

N/A

Do you have any faculty members who have clinical time dedicated to adult sleep

medicine/surgery?

Yes

No

N/A

How many faculty members have clinical time dedicated to adult sleep medicine/surgery at

your institution?

0

1

2

3

4

5

>6

N/A

Of those faculty members who have dedicated time to adult sleep medicine/surgery, what

percentage of their work time do they spend on sleep medicine or sleep surgery?

100%

75%-99%

50%-74%

25%-49%

<25% time

We do not have sleep medicine/surgery faculty

N/A

No. of faculty members who are board certified in sleep medicine by the American Board of

Medical Specialties and involved in training of Otolaryngology residents:

0

1

2

>3

N/A

Do those faculty members with a practice dedicated to sleep medicine/surgery, share their

practice time with other specialties?

Yes

No

We do not have sleep medicine/surgery faculty

N/A

If yes which specialties (multiple answers allowed)

None (ie I did not answer yes to the previous question)

General ENT

Rhinology

Otology

Head and neck surgery

Laryngology

Pediatric ENT

Oral and Maxillary Facial Surgery

Facial plastic surgery

Allergy

Other

N/A

Number of total months residents have exposure to adult sleep surgery or sleep clinic

throughout residency (type N/A if not applicable) : ____ months

Estimated percentage of time spent by residents on adult sleep surgery or sleep clinic during

those months (type N/A if not applicable): ____%

Adult sleep surgery procedures that residents perform during their training at your institution

(choose all that apply)

Tonsillectomy

Adenoidectomy

Septoplasty

Septoplasty/rhinoplasty

Nasal valve surgery

Turbinate reduction

Traditional Uvulopalatopharyngoplasty (UPPP)

Tongue suspension

Hyoid suspension

Partial glossectomy/tongue based resection

Geniotubercle advancement

Maxillomandibular advancement (MMA)

Hypoglossal nerve stimulator

Palatal expansion

Modified UPPP

Other : _____

N/A

Do residents receive education in interpreting in-lab polysomnogram results?

Report only

Original data only

Original data and report

None

N/A

Do residents receive education in interpreting home sleep study results?

Report only

Original data only

Original data and report

None

N/A

Do resident receive training in analyzing the compliance and the efficacy data from

continuous positive airway pressure devices

yes

no

unknown

N/A

How many residents have been accepted into a sleep surgery fellowship from your

Otolaryngology residency program in the past 10 years?

0

1

2

3

4

5

6

7

8

9

10

Greater than 10

N/A

How many residents have been accepted into an ACGME accredited sleep fellowship

following Otolaryngology residency in the last 10 years? ___________

0

1

2

3

4

5

6

7

8

9

10

Greater than 10

N/A

How Satisfied Overall Are You With Your Program's Resident Exposure and

Education in Sleep Medicine/Surgery?

Extremely

Very

Fairly

Poorly

Questions comment or concerns can be included here: _________
